# Anomalies du spermogramme en consultations prénuptiales et dans les couples infertiles à Butembo, République Démocratique du Congo

**DOI:** 10.11604/pamj.2020.37.155.25380

**Published:** 2020-10-13

**Authors:** Philemon Matumo, Gabriel Bunduki, Idephonse Soly Kamwira, Juakali Sihalikyolo, Katenga Bosunga

**Affiliations:** 1 Département de Gynécologie et Obstétrique, Faculté de Médecine, Université Catholique du Graben, Butembo, République Démocratique du Congo,; 2 Département de Maladies infectieuses, Université Catholique du Graben, Butembo, République Démocratique du Congo,; 3 Faculté de Pharmacie, Université Catholique du Graben, Butembo, République Démocratique du Congo,; 4 Département de Gynécologie et Obstétrique, Faculté de Médecine et de Pharmacie, Université de Kisangani, Kisangani, République Démocratique du Congo

**Keywords:** Infertilité masculine, spermogramme, spermocytogramme, oligoasthénotératozoospermie, Butembo, Male infertility, spermogram, spermocyogram, oligoasthenoteratozoospermia, Butembo

## Abstract

La présente étude visait à déterminer la prévalence des anomalies du spermogramme au cours des consultations prénuptiales et chez les hommes des couples infertiles à Butembo. C´est une étude rétrospective descriptive effectuée au Centre Universitaire de Diagnostic du Graben. La population d´étude était constituée de 890 sujets masculins de 21 à 57 ans d´âges dont 779 sujets pour les examens prénuptiaux et 111 sujets pour tests de fertilité. La prévalence globale des anomalies du spermogramme dans cette population est de 25.8% soit une incidence de 22.9% des consultants prénuptiaux et 46.0% d´incidence des hommes des couples infertiles. Le pH moyen était de: 7.22 ± 0.22. Le volume moyen de sperme recueilli était: 2.56 ± 1.41 ml. Les anomalies ont porté sur tous les paramètres du spermogramme avec prédominance d´association d´anomalies chez le même sujet à 86.5% avec en tête l´oligoasthénotératozoospermie dans 44.8% des cas. Pris isolément, on retrouve au premier rang l´asthénozoospermie chez 90.9% des cas suivie de l´oligozoospermie chez 87.4% des cas, la tératozoospermie chez 66.9% des cas, la nécrozoospermie chez 55.6% des cas et l´azoospermie chez 10.4% des cas. A l´issue de cette étude, il ressort que le profil cytologique du spermogramme de cette population de Butembo est dominé par des associations d´anomalies. Ceci demande donc la réalisation simultanée d´autres tests tels que les examens bactériologiques et les dosages de marqueurs biochimiques, afin d´identifier les étiologies et d´y apporter les thérapeutiques appropriées.

## Abstract (en)

The purpose of this study was to determine the prevalence of spermogram abnormalities in men consulting for premarital screening and in infertile couples in Butembo. We conducted a retrospective descriptive study at the Graben University Diagnostic Center. The study population consisted of 890 male subjects aged 21-57 years, of whom 779 underwent prenuptial screening and 111 subjects fertility tests. The overall prevalence of spermogram anomalies in this population was 25.8%, corresponding to a total incidence of 22.9% of premarital consultants and 46,0% of males in infertile couples. Mean pH was: 7.22+/- 0.22. The average volume of semen collected was: 2.56 +/- 1.41 ml. Abnormalities were detected in all spermogram parameters, with a predominance of abnormalities in the same subject (86.5%), mainly in oligoasthenoteratozoospermia (44.8% of cases). Asthenozoospermia was the most common abnormality (90.9% of cases), followed by oligozoospermia (87.4% of cases), teratozoospermia (66.9% of cases), necrozoospermia (55.6% of cases), and azoospermia (10.4% of cases). This study highlights that the cytological profile of the spermogram of this population in Butembo is dominated by associations of anomalies. Other tests such as bacteriological examinations and biochemical marker assays are necessary, in order to identify the causes of abnormalities and to provide appropriate therapies.

## Introduction

Le spermogramme-spermocytogramme est un examen de base dans l´évaluation qualitative et quantitative du sperme [1-3]. Le spermogramme désigne tous les tests réalisés à l´état frais tandis que le spermocytogramme désigne l´analyse cytologique faite dans un second temps à partir d´un frottis coloré du sperme [1, 3]. Le spermogramme peut montrer plusieurs anomalies qui ne sont toutefois pas forcément synonyme d´infertilité masculine. Néanmoins, l´augmentation des anomalies du sperme, a été considérée comme cause possible de la baisse de la fertilité chez les hommes [4].

En effet, l´éjaculat normal contient des spermatozoïdes présentant des variations importantes de la taille et de la forme de la tête, de l'acrosome, de la pièce intermédiaire et du flagelle. Cette hétérogénéité s'explique par le fait que le spermatozoïde, cette cellule hautement différenciée, est le résultat ultime d'un processus complexe: la spermatogenèse [5]. Ce processus peut être la cible de facteurs toxiques endogènes ou exogènes qui peuvent induire la production excessive de spermatozoïdes morphologiquement anormaux [5]. Les paramètres du sperme tels que: pH, mobilité, morphologie et vitalité sont reconnus comme jouant un rôle important dans la compétence fonctionnelle des spermatozoïdes [4]. Ce qui justifie l´importance du spermogramme dans l´orientation diagnostique de l´infertilité du couple.

Ces problèmes d´infertilité touchent environ 1 couple sur 10 dans le monde, voir 1 sur 7 couples dans certaines régions et l´on assiste à une baisse de la fertilité au fil des années [3, 6]. Cette situation préoccupe les couples concernés et, souvent, les déstabilise, surtout dans les régions africaines. C´est donc un problème de santé publique dans les pays en voie de développement du fait de sa prévalence, de la généralisation de sa répartition et des difficultés inhérentes à sa prise en charge [7]. Il y a quelque temps, la femme était considérée comme la seule responsable de l´infertilité du couple [3]. De nos jours, l´on se rend compte que les deux sexes sont touchés [6]. Ainsi, on retrouve 30% de causes masculines, 30% de causes féminines, et 30% de causes mixtes et aucune cause n´a été retrouvée dans le reste des cas [6, 7]. Ceci montre la part de responsabilité de l´homme qui pendant longtemps, dans le contexte africain, a indexé la femme comme l´unique responsable.

De nombreuses études ont objectivé une altération de la qualité de sperme au cours des dernières décennies avec, sans doute, une implication de l´environnement [8]. Aussi, plusieurs médicaments couramment utilisés et des drogues récréatives ou illicites ont un effet néfaste sur la fertilité de l´homme [9]. Particulièrement à l´Est de la République Démocratique du Congo, la rapide croissance démographique, l´urbanisation accrue, l´exploitation incontrôlée des ressources naturelles et des explosions des armes de guerre auraient généré une source de pollution ces dernières années. Cette situation présente un réel danger pour l´homme du fait des phénomènes de bioaccumulation dans la chaîne alimentaire et dans l´air ambiant, ce qui se répercuterait sur sa fertilité. Cependant, dans notre contexte de Butembo le dépistage des anomalies du spermogramme n´est pas encore fait. Le présent travail vise d´appréhender l´infertilité masculine dans notre milieu par des analyses du sperme. Ainsi, le but de cette étude était de déterminer la prévalence des anomalies du spermogramme au cours des consultations prénuptiales et chez les hommes ayant consultés le Centre Universitaire de Diagnostic au Graben (CUDG) pour exploration des causes de l´infertilité.

## Méthodes

Il s´agit d´une étude rétrospective descriptive effectuée au Centre Universitaire de Diagnostic au Graben (CUDG) de Janvier 2014 à Décembre 2018. Le CUDG se trouve dans la ville de Butembo, à l´Est de la République Démocratique du Congo. Il s´agit d´un centre de recherche biomédicale apparenté à l´Université Catholique du Graben. Les données ont été recueillies à partir des dossiers des examinés au CUDG. La population d´étude était constituée de tous les hommes envoyés au CUDG pendant la période d´étude soit pour dépistage dans le cadre de leurs consultations prénuptiales soit pour bilan de fertilité pour les couples infertiles. L´échantillonnage était non probabiliste de convenance. L´échantillon de sperme était prélevé au CUDG. En effet, le sperme était recueilli au laboratoire par masturbation dans un flacon à large goulot chez des sujets ayant observé une abstinence de 3 à 5 jours selon le protocole de l´Organisation Mondiale de la Santé (OMS) version 2010.

Les paramètres étudiés étaient l´âge du sujet et les paramètres d´analyse du sperme comme le volume, le pH, la concentration des spermatozoïdes, la mobilité, la morphologie et la vitalité. Les résultats obtenus ont été classés selon les critères de définition des anomalies du sperme et d´évaluation du spermogramme définis par l´Organisation Mondiale de la Santé (OMS) version 2010 (Tableau 1) [3]. Ensuite, la qualité du sperme a été classée en normal, probablement normal, probablement anormal et anormal suivant les normes en vigueur pour une appréciation détaillée du sperme selon Briex (Tableau 2) [10]. Les données ont été saisies et traitées à l´aide du logiciel Epi-info version 6.04.fr.

**Tableau 1:** critères de définition des anomalies selon l’OMS 2010

Paramètres du sperme	Normes OMS 2010	Définition de l’anomalie
Volume du sperme	≥1,5 ml	<1,5 ml: hypospermie
		>6 ml: hyperspermie
pH du sperme	7,2 – 8	
Numération des spermatozoïdes	≥15 millions/ml	0: azoospermia
		<15 millions/ml: oligospermie
		>200 millions/ml: polyspermie
Mobilité	≥40% de Mobilité totale (a+b+c)	
	≥32% de mobilité progressive (a+b)	≤32% de mobilité progressive: asthénospermie
Morphologie	>4% selon Kruger	≤4%: tératospermie
Vitalité	>58% de formes vivantes	≤58%: necrospermie
Leucocytes	<1 millions/ml	≥1 millions: leucospermie

**Tableau 2:** critères d’évaluation de la qualité du sperme (BRIEX)

	Volume (ml)	Concentration (millions/ml)	Formes vivantes (%)	Formes mobiles (%)	Formes anormales (%)
Normal	2 – 5	40 – 200	>80	>80	<30
Probablement normal	>2	20 – 40	70 – 80	60 – 80	30 – 50
	<5 – 7				
Probablement anormal	1,5 – 2	15 – 20	50 – 70	40 – 60	50 – 80
	>5				
Anormal	>7	<15	<5	<40	>80
Spermie	hypo-	azoo-	ecro-	asthéno-	térato-
	hyper-	oligo-			
		polyzoo-			

## Résultats

Nous avons colligé 890 dossiers des sujets ayant fait le spermogramme au CUDG pendant notre période d´étude dont 779 sujets pour les examens prénuptiaux et 111 sujets pour tests de fertilité des hommes des couples infertiles. La prévalence globale des anomalies du spermogramme dans cette population est de 25,8% soit 230 spermogrammes anormaux sur 890 répartis comme suit: 179 cas des 779 spermogrammes des consultants prénuptiaux soit une incidence de 22,9% et 51 cas des 111 spermogrammes des hommes des couples infertiles soit une incidence de 46,0%. Les patients étaient âgés de 21 à 57 ans, l´âge moyen était de 31 ans avec une population plus jeunes consultants pour les examens prénuptiaux que celle consultants pour le test de fertilité (Tableau 3). En rapport avec les anomalies, le spermogramme était pathologique dans 22,9% de cas dans la population des consultants prénuptiaux et 46,0% de cas chez les hommes des couples infertiles. Le pH était < à 7,2 dans 179 cas soit 20,1%; le pH le plus élevé concernait 3 patients soit 0,4% et était de 9,0; le pH le plus bas était de 6,4 et concernait 6 patients soit 0,8% des cas. Le pH moyen était de: 7,22 ± 0,22. Le volume de sperme était inférieur à 1,5 ml dans 183 cas soit 20,6%, et supérieur à 5 ml dans 17 cas soit 1,9%. Le plus faible volume de sperme était de 0,3 ml, et était retrouvé dans 8 cas soit 0,9%; le volume de sperme le plus important était de 7,8 ml et était retrouvé dans 1 cas (0,1%). Le volume moyen de sperme recueilli: 2,56 ± 1,41 ml. Dans cette même série, on retrouve l´asthénozoospermie dans 90,9% des cas, l´oligozoospermie dans 87,4% des cas, la tératozoospermie dans 66,9% des cas, la nécrozoospermie dans 55,6% des cas et l´azoospermie dans 10,4% des cas de cet échantillon (Tableau 4). Dans 86,5% des cas les anomalies du spermogramme associent anomalies de qualité et de quantité des spermatozoïdes, telles qu´oligoasthénotérato zoospermie, asthénotérato-zoospermie, et oligoasthénozoospermie (Tableau 5).

**Tableau 3:** répartition des consultants prénuptiaux et des hommes des couples infertiles selon leur âge

Tranches d’ âge (ans)	Consultants prénuptiaux		Hommes des couples infertiles	
	n (N=779)	Cas (%) N=179	n (N=111)	Cas (%) N=51
<25	221	49 (22,2)	6	1 (16,7)
25 – 34	458	103 (22,5)	48	16 (33,3)
35 – 44	87	22 (25,3)	32	19 (59,4)
≥45	13	5 (38,5)	25	15 (60,0)

**Tableau 4:** répartition des anomalies en fonction de la qualité du sperme de la population globale

Qualité du sperme N= 890	Volume n (%)	Concentration n (%)	Formes vivante n (%)	Formes mobiles n (%)	Formes anormales n (%)
Normal	493 (55,4)	511 (57,4)	659 (74,0)	566 (63,6)	564 (60,0)
Probablement normal	172 (19,3)	149 (16,7)	103 (11,6)	115 (12,9)	172 (18,2)
Probablement anormal	42 (4,7)	63 (7,1)	55 (6,2)	41 (4,6)	53 (6,0)
Anormal	183 (20,6)	167 (18,8)	73 (8,2)	168 (18,9)	101(15,8)
Spermie N=230	Hypo- 208 (90,4)	Azoo- 24 (10.4) Oligo- 201 (87,4)	Necro- 128 (55,6)	Astheno- 209 (90,9)	Terato- 154 (66,9)
	Hyper- 17 (7,4)	Polyzoo- 5 (2,2)			

**Tableau 5:** répartition des patients en fonction de l’association d’anomalies de qualité et de quantité des spermatozoïdes

Anomalies associées (N=230)	n	%
Oligoasthénotératozoospermie	103	44,8
Oligoasthénozoospermie	84	36,5
Asthénotératozoospermie	12	5,2

## Discussion

Considérant les conditions de travail dans les pays en voie de développement, le spermogramme reste l´examen clé pour explorer la fertilité masculine. Cependant, sa sensibilité et sa spécificité dans l´approche diagnostique restent modérées, rappelant les éventuelles limites de son interprétation. Mais il constitue la première étape dans l´identification des facteurs en cause de l´infertilité masculine [3, 4, 11]. Cette analyse, simple à première vue, présente de réelles difficultés avec pour conséquence une fiabilité relative des résultats d'un laboratoire à l'autre du fait du caractère subjectif de l'analyse microscopique de la morphologie des spermatozoïdes humains qui dépend principalement des mécanismes de la vision et de son intégration au niveau cérébral [12,13]. Dans notre série la prévalence des anomalies du spermogramme a été différente selon qu´il s´agit du dépistage par test de fertilité soit 22,9% des cas ou qu´il s´agit d´une population présélectionnée des hommes des couples infertiles soit 46,0% des cas. La quasi-totalité des études dans la littérature se sont intéressées uniquement à la prévalence dans la population des hommes consultant pour infertilité dans leur couple. Le résultat de notre série est légèrement inférieur à celui trouvé par Juakali SKV en 2015 dans la ville de Kisangani en RDC (soit 57,1%) [14]. En effet, la différence climatique pourrait y être pour quelque chose avec un climat quasi chaud à Kisangani. On note aussi que ce dernier avait utilisé les critères d´évaluation différents (OMS 1999) tout comme tous les autres auteurs d´avant 2010. Il est de loin inférieur à ceux trouvés par la plupart d´auteurs du nord: Hounnasso en 2013 au Cotonou (76,5%) [2], Xu Ty en 2011 en Chine (72,6%) [15], Sakande en 2012 à Ougadougou (84,1%) [16].

Cette appartenance au groupe des couples infertiles est un facteur prédictif d´anomalies du spermogramme. Ce qui justifie cette différence de fréquence de ces anomalies dans les deux populations. Mais devant le développement des moyens diagnostiques et thérapeutiques, le clinicien se verra de plus en plus sollicité par les patients infertiles. La plupart des études s´accordent à dire qu´au cours des dernières décades, on note un déclin remarquable du taux de fertilité dans le monde et beaucoup plus dans les pays du nord [17]. La moyenne d´âge de la population étudiée était de 31 ans avec des extrêmes de 21 et 57 ans. La majorité de la population était constituée par la tranche d´âge de 25 à 34 ans soit 506 sujets suivi de moins de 25 ans soit 227 sujets. Ceci reflète l´âge auquel le jeune homme se marie dans notre société et c´est la période d´activité génitale par excellence où l´homme accorde beaucoup plus d´intérêt à sa santé reproductive [14, 16]. Mais le Tableau 4 monte une incidence croissante d´anomalies du spermogramme selon l´âge: 22,0% pour les moins de 25 ans; 23,5% entre 25 et 34 ans; 34,4% entre 35 et 44 ans et 55,6% de 45 ans et plus. Ceci a déjà été prouvé par d´autres auteurs dans la littérature [2, 6, 8]. Juakali à Kisangani quand bien même avec les critères OMS 1999 avait trouvé aussi que la fréquence des anomalies du spermogramme augmente avec l´âge des sujets allant de 49,1% pour les sujets de moins de 30 ans à 66,7% pour ceux d´âge supérieur à 50 ans [14]. Certains facteurs socioéconomiques font que le mariage se fait de plus en plus tardivement. Pendant ce temps, le vieillissement biologique diminue le potentiel de fécondité des individus, d´où les difficultés à concevoir des couples de nos jours [17]. Fréderic P [18] dans son analyse biodémographique sur l´âge et l´infertilité masculine précise que cette baisse de la fertilité de l´homme n´est pas celle du déclin de la fécondabilité de la semence, mais bien celle de la capacité à procréer (engendrer une naissance vivante) en l´absence de contraintes autres que celles du vieillissement de l´homme et la dysfonction érectile ou la simple baisse de libido ne sont pas étrangères à ce vieillissement.

Dans notre étude, les anomalies ont porté sur tous les paramètres du spermogramme avec prédominance d´association d´anomalies chez le même sujet à 86,5% avec en tête l´oligoasthénotératozoospermie (OATS) dans 44,8% des cas suivi de l´oligoasthénozoospermie dans 36,5% et enfin l´asthénotératozoospermie dans 5,2% des cas sans prendre en compte les anomalies de volume ni la nécrozoospermie. Ces dernières prises isolement ont concerné respectivement 90,4% et 55,6% des cas. La répartition des anomalies du spermogramme prises isolement se rapproche de celles de la plupart des auteurs dans la littérature même si différemment appréciée [2, 5, 14, 15, 17]. Dans cette série, on retrouve au premier rang l´asthénozoospermie (90,9% des cas) suivie de l´oligozoospermie (87,4% des cas), la tératozoospermie (66,9% des cas) et la nécrozoospermie (55,6% des cas). Il est à noter que l´azoospermie occupe également une part importante dans l´infertilité avec 10,4% des cas dans notre échantillon. Cette incidence élevée de l´asthénozoospermie reflète la gravité de l´hypofertilité masculine dans notre société car la mobilité des spermatozoïdes est le critère prédictif le plus important pour la survenue d´une grossesse spontanée [1]. Ainsi, d´un point de vue diagnostic, une anomalie de la mobilité peut correspondre à une anomalie de structure des spermatozoïdes ou à des anomalies de leur maturation lors du transport dans les voies génitales. Ce qui justifie le plus souvent les associations d´anomalies telles que observées dans cette étude et la part du dosage des marqueurs biochimiques du plasma séminal dans la recherche d´étiologie topographique.

En rapport avec le déclin des paramètres spermatiques sur les dernières décennies, la question de l´impact de l´environnement sur la santé reproductive constitue une préoccupation grandissante dans les pays industrialisés [8, 19]. Honoré M Gbetoh *et al*. à Cotonou soupçonnent que la présence de cadmium dans le sperme, même à un taux infirme, serait responsable des anomalies du spermogramme [20]. Richard Mutshimbe Mukendi *et al*. en République Démocratique du Congo montre bien d'une part des fortes concentrations urinaires d'arsenic et de cadmium et d'autre part l'altération plus rapide et plus sévère des éléments du spermogramme chez les hommes vivants en zone minière, suggérant une baisse de la fertilité masculine [21]. Dans l´étude de Juakali à Kisangani, le facteur incriminé était le réchauffement quasi permanent des testicules des «tolekistes» qui entravait ainsi le bon déroulement de la spermatogenèse [14]. D´autres auteurs en fin incriminent les effets du stress oxydatifs et de la pollution environnementale [8, 17, 22, 23]. Leurs sources sont liées au mode de vie de l´homme contemporain (tabac, drogues, bains chauds, expositions aux perturbateurs endocriniens) et aux pathologies génératrices de radicaux libres oxygénés (varicocèle, infection des glandes accessoires masculines particulièrement les infections sexuellement transmissibles) [22, 23].

Les dérivés actifs de l´oxygène (en anglais ROS pour reactive oxygen species) sont des produits physiologiques du métabolisme cellulaire qui peuvent devenir délétères pour de nombreuses cellules, dont les spermatozoïdes s´ils augmentent dans l´environnement de la cellule, soit du fait d´une augmentation de leur production, soit d´un défaut de dégradation ou d´élimination [22]. Les spermatozoïdes, comme toute cellule vivante, se défendent contre ce stress. Ces cellules sont équipées de systèmes antioxydants tels que les enzymes antioxydantes (superoxyde, dismutase, glutathion peroxydase) et les éléments trace (zinc, sélénium). Ces derniers semblent jouer un rôle très important dans la fertilité masculine car ils constituent des cofacteurs des enzymes antioxydantes et interviennent dans l´intégrité structurale des spermatozoïdes. Ainsi, les spermatozoïdes, soumis à un stress oxydatif, présentent des altérations structurales de l´ADN. Ces radicaux libres peuvent aussi induire une peroxydation des lipides membranaires des spermatozoïdes, ce qui augmente les anomalies dans la pièce intermédiaire, avec pour conséquence une diminution de la mobilité du spermatozoïde [24, 25]. L´impact du stress oxydatif sur le spermatozoïde pourrait également aboutir à une baisse du pouvoir fécondant du spermatozoïde et du développement embryonnaire [23]. Ceci justifie aujourd´hui l´adjonction à l´arsenal thérapeutique de l´infertilité masculine la prescription de traitements antioxydants, notamment en cas d´oligoasthénotératospermie idiopathique et chez les hommes pris en charge en Assistance Médicale à la Procréation (AMP) [22].

## Conclusion

Cette étude menée à Butembo durant une période de 5 ans montre une prévalence d´anomalies du spermogramme à 22,9% dans la population masculine générale et de 46,0% chez les hommes des couples incapables de concevoir spontanément. Le profil cytologique du spermogramme de cette population affectée est dominé par des associations d´anomalies avec prédominance de l´oligoasthénotératozoospermie. Ceci justifierait la prescription simultanée du spermogramme et d´autres examens tels que le prélèvement pour examens bactériologiques et les dosages de marqueurs biochimiques, afin d´identifier les étiologies de l´infertilité masculine, et d´y apporter les thérapeutiques les plus appropriées.

### Etat des connaissances sur le sujet

- Déclin de la fécondité de l´homme avec les évènements de changements climatiques, la civilisations rapides par tout au monde avec l´avènements des produits chimiques qui altèrent la spermatogenèse;
- La prévalence et répartition des anomalies du spermogramme dans différentes régions du monde.

### Contribution de notre étude à la connaissance

- La comparaison de la prévalence et répartition des anomalies du spermogramme chez les jeunes en consultations prénuptiales et dans les couples infertiles;
- L´utilisation des autres critères combinant les directives de l´OMS pour juger de la qualité du sperme.
